# Comparing the Dietary Habits and the Food Choices Between Italian and Dominican Adult Populations: Focus on Fruit and Vegetable Intakes and Their Association with Skin Carotenoid Levels

**DOI:** 10.3390/foods13203323

**Published:** 2024-10-19

**Authors:** Giuseppina Augimeri, Manuel Soto, Fabrizio Ceraudo, Giovanna Caparello, Melisa Villegas Figueroa, Mirko Cesario, Lorenzo S. Caputi, Berniza Calderon, Daniela Bonofiglio

**Affiliations:** 1 Department of Pharmacy, Health and Nutritional Sciences, University of Calabria, Arcavacata di Rende (CS), 87036 Rende, Italy; giuseppina.augimeri@unical.it (G.A.); fabrizio.cer96@gmail.com (F.C.); caparello.giovanna@gmail.com (G.C.); mirkocesario@gmail.com (M.C.); 2 Research Unit, Centro Médico de Diabetes, Obesidad y Especialidades (CEMDOE), Clara María Pardo Street, Santo Domingo 10135, Dominican Republic; manuel.soto@cemdoe.com (M.S.); berniza.calderon@intec.edu.do (B.C.); 3 School of Medicine, Instituto Tecnológico de Santo Domingo (INTEC), Los Proceres Avenue, Santo Domingo 10602, Dominican Republic; amelisavillegas@gmail.com; 4 UNICARIBE Research Center, University of Calabria, 87036 Rende, Italy; lorenzo.caputi@fis.unical.it; 5 Surface Nanoscience Group, Department of Physics, University of Calabria, 87036 Rende, Italy; 6 Sociedad Dominicana de Endocrinología y Nutrición (SODENN), 157 Independencia Avenue, GS Professional Building, Santo Domingo 10206, Dominican Republic; 7 Centro Sanitario, University of Calabria, Via P. Bucci, Arcavacata Di Rende (CS), 87036 Rende, Italy

**Keywords:** eating habits, fruit and vegetable intake, Veggie Meter^®^, Mediterranean diet, carotenoid score

## Abstract

The Mediterranean Diet (MD) is characterized by a high intake of fruits and vegetables (FVs), which is considered as an important contributor to the beneficial effects of the MD pattern. In this cross-sectional study, we compared the food choices, evaluated by dietary habit questionnaires, of a sample of 995 adults, including 601 and 394 participants from Southern Italy and the Dominican Republic, respectively. In addition, we focused on their FV consumption, assessed by the Mediterranean Diet Adherence Screener (MEDAS) questionnaire, and on its association with skin carotenoid levels as measured by the Veggie Meter^®^. We found that a significantly higher percentage of Italians had five meals/day and breakfast compared to Dominicans (five meals/day: 43 vs. 25, *p* < 0.05; breakfast: 89 vs. 79, *p* < 0.05), whereas a lower percentage of participants from Italy consumed snacks between the two meals compared to the Dominican Republic population (47 vs. 70, *p* < 0.005). Most of the participants from both populations had breakfast at home. However, 59.3% of Italians and 27.5% of Dominicans (*p* = 0.005) had breakfast between 7:00 and 9:00 a.m., whereas 5.8% and 27.5% (*p* = 0.001) had breakfast after 9:00 a.m., respectively. Milk/yogurt and eggs were the most consumed foods for breakfast in Italy and the Dominican Republic, respectively. Regarding the main meals, most of the Italians and Dominicans had a first course for lunch and a second course for dinner. Of note, we observed that approximately half of the Italians ate FVs in their main meals and had a higher carotenoid score than the Dominicans. Interestingly, in the multiple linear regression analysis, we found that the carotenoid score was positively associated with sex (β = 0.078; *p* = 0.009), age (β = *0.008*; *p* = 0.001), vegetable consumption (β = 0.12; *p* = 0.041) and the perception of a healthy diet (β = 0.12; *p* = 0.001) in the Dominic Republic population, while the carotenoid score was directly associated with sex (β = 54.97; *p* < 0.0001) and both vegetable (β = 25.42; *p* = 0.0008) and fruit (β = 38.61; *p* < 0.0001) consumption in the Italian sample. Our findings confirm the need to promote nutrition-based interventions to encourage FV intake, particularly in non-Mediterranean countries.

## 1. Introduction

Globalization and urbanization have changed the dietary and lifestyle patterns of many communities around the world. Ultra-processed foods, sugar-sweetened beverages and foods with a high saturated fat content, like fast food, are now more easily obtained and consumed. These changes have contributed to the global increase in metabolic and chronic diseases such as obesity, type 2 diabetes mellitus, cardiovascular diseases and cancer [1,2,3,4].

It is widely known that the consumption of a healthy diet can help prevent multiple chronic conditions [5]. A comprehensive review of the literature suggests that plant-based diets, low-fat and carbohydrate-restricted diets, the DASH (Dietary Approaches to Stop Hypertension) diet and time-restricted diets represent the most extensively investigated diets for their potential beneficial effects on health [6]. Among plant-based diets, the Mediterranean Diet (MD) pattern has been associated with multiple health benefits, including the prevention of obesity, diabetes mellitus, metabolic syndrome, cardiovascular diseases and cancer, and the reduction in overall mortality [7,8], making it an effective health strategy. This dietary model takes its name from the geographic region from which it emerged, appearing first in the population living in Mediterranean countries, including Spain, Morocco, Egypt, Greece and Southern Italy. It is characterized by the high intake of fruits and vegetables (FVs), legumes, cereals, olive oil, minimally processed foods, poultry and fish [7,8,9].

The high intake of FVs in the MD is one of the main factors that is considered to contribute to its beneficial effects [10,11]. Most FVs have a high content of carotenoids, which are colored organic pigments that are produced by plants. These pigments are known for their anti-oxidative and anti-inflammatory effects, and have been linked to a reduced risk of cardiovascular diseases [12,13,14]. Recent studies have shown that the measurements of skin carotenoids offer an indirect measurement of serum carotenoid levels [15,16]. This has made it possible to estimate carotenoid levels through the use of light spectroscopy, a non-invasive pressure-based method performed with the Veggie Meter^®^, which has been validated as an objective measurement method in previous studies [17,18].

Adherence to the MD, which can be measured through the administration of validated food questionnaires like the Mediterranean Diet Adherence Screener (MEDAS), has been associated with higher levels of skin carotenoids, as is expected by the increased consumption of FVs [19,20]. However, adherence to the MD and the consumption of FVs varies greatly according to geographic regions. Even in Mediterranean countries like Italy, the dietary patterns vary across regions, with urban areas showing reduced adherence to the MD [21]. Furthermore, statistics from the European Union report that more than 30% of persons aged 15 and older ate zero portions of FVs daily in 2019, while, in Italy, the proportion reported was 23.8% [22]. Evidence about the adherence to the MD pattern in the region of Latin America and the Caribbean is limited. However, there is information available about the consumption of FVs, which is the cornerstone of the MD. In the Dominican Republic, the National Demographic and Health Survey reported that only around 83% of adults consume FVs at least 2–3 days in a week [23].

Considering all of the aforementioned, and the United Nations’ sustainable development to promote well-being for all, health promotion strategies that encourage healthy diets are essential to help reduce the burden of chronic diseases. To create effective strategies, research on different dietary patterns and their association to health outcomes and indicators in communities of different geographical regions are needed. 

The aim of this study was to evaluate the eating habits of a sample population of 995 adults from Southern Italy and from the Dominican Republic, focusing on FV consumption and its association with skin carotenoid levels as measured with the Veggie Meter^®^.

## 2. Materials and Methods

### 2.1. Study Design

This is a cross-sectional study involving 995 adults, including 601 and 394 participants from the University of Calabria, Italy, and from the Instituto Tecnológico de Santo Domingo (INTEC) and the Centro Médico de Diabetes, Obesidad y Especialidades (CEMDOE), Dominican Republic, respectively. Data collection was performed from September 2023 to October 2023. Included participants were 18 years of age or older, had Italian or Dominican nationality and gave informed consent for participation. Subjects with liver diseases were excluded from the study [19].

This study, part of the UNICARIBE project, which aims to develop joint research programs between the University of Calabria in Italy and Caribbean research institutions, was approved by the Italian Ethics Committee (#67342/2023) and by the Ethics Committee of the Instituto Tecnológico de Santo Domingo in the Dominican Republic.

### 2.2. Sociodemographic and Anthopometric Measurments

Participants were asked to complete a survey to collect general and sociodemographic data. Weight and height were collected by using a previously calibrated analog scale [19]. All of the anthropometric measurements were performed by trained personnel. BMI was calculated using the standard formula.

### 2.3. Dietary Habits Assessment

Participants completed a questionnaire on dietary habits, including information on the usual content of their meals for lunch, dinner and snacks, as previously reported [24]. Additionally, the usual time and location of breakfast was gathered. FV consumption was assessed using the MEDAS questionnaire [19]. The MEDAS questionnaire has been validated for use in both Mediterranean and non-Mediterranean countries [20,25].

### 2.4. Skin Carotenoid Levels

Skin carotenoid levels were measured using the Veggie Meter^®^, which uses light spectroscopy. The device was properly calibrated and measurements were performed according to the manufacturer’s instructions. The scores ranged from 0 to 800, with higher scores being indicative of a higher skin carotenoid concentration [17].

### 2.5. Sample Size and Power Calculation

A minimum sample size of 385 participants per country was calculated based on a 95% confidence interval, a margin of error and an alpha value of 5%. As a result of a high participation rate, 601 and 394 Italian and Dominican participants, respectively, were recruited. Post hoc power calculations yielded a statistical power (β) of 86% for difference in means between the populations and small effect sizes (Cohen’s D = 0.02).

### 2.6. Statistical Analysis

Quantitative variables were reported as the mean and standard deviation (SD), while categorical variables were reported with the absolute frequency and percentage. Central limit theorem and normal distribution were assumed considering the large sample size. For this reason, Student’s *t*-Test and chi-squared tests were used to assess statistically significant differences between the Italian and Dominican Republic populations, where applicable, and *p*-values lower than 0.05 were considered as statistically significant.

To determine the variables associated with skin carotenoid levels, multiple linear regression models using the Ordinal Least Squares (OLS) method were created for both the Italian and Dominican populations. For the linear regression model of the Dominican population, the skin carotenoid levels were log-transformed to comply with the linear regression models’ assumptions. Statistical analyses were performed using the GraphPad-Prism 9 and the Jupyter Notebook version 8.6.0 software programs.

## 3. Results

### 3.1. Characteristics of Study Population 

This study included a cohort of 995 participants previously enrolled in the UNICARIBE project [19]. The sample population consisted of 601 and 394 participants recruited from Southern Italy and the Dominican Republic, respectively. Table 1 summarizes the anthropometric and general characteristics of the study population. The Italian population had a lower mean BMI, as previously reported [19]. Categorizing the population by BMI values, significant differences were observed between Italian and Dominican women (*p* < 0.0002) (Supplementary Table S1). Furthermore, the smoking rate was higher among Italians (24.8 vs. 8.1, *p* = 0.001), as well as the frequency of biochemistry analysis over 12 months (75.7 vs. 49.7, *p* = 0.0001) (Table 1), in both sexes (Supplementary Table S1). In addition, the iodine salt intake (83.2 vs. 69.3, *p* = 0.02) was higher in Italians (Table 1), with a notably higher percentage of men in Italy consuming iodine salt compared to Dominicans (Supplementary Table S1). No significant differences were observed in the habits of periodically checking the arterial pressure and in the participants’ perceived intake of healthy food. 

### 3.2. Percentage of Participants Consuming Breakfast, Meals and Snacks in the Italian and Dominican Republic Populations

**Figure 1:**
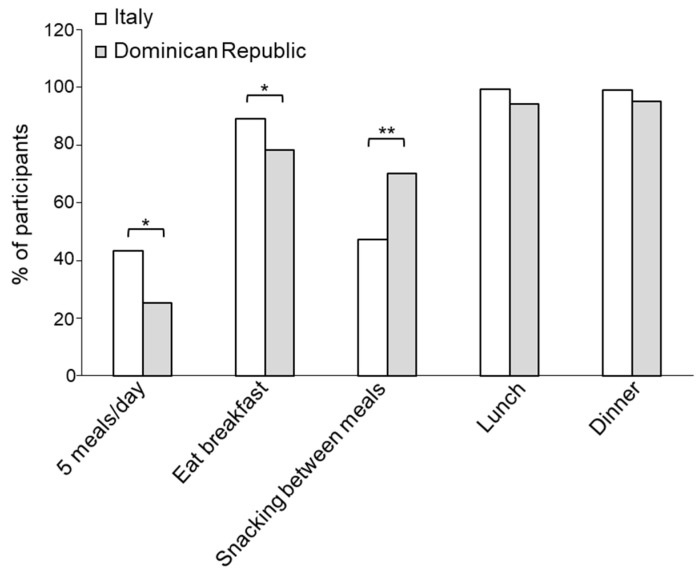
Distribution of the participants from Italy and the Dominican Republic based on their eating occasions. * *p* < 0.05; ** *p* < 0.005.

The MD guidelines suggest having five meals/day, including breakfast, snacks between the main meals, lunch and dinner. Investigating the dietary habits in terms of meal frequency, we observed a significantly higher percentage of Italians reporting having five meals/day compared to Dominicans (43 vs. 25, *p* < 0.05) (Figure 1). Categorizing the population by sex, we observed a significantly higher percentage of Italian women consuming five meals/day when compared to Dominican women (Supplementary Table S2). Moreover, the percentage of Italians having breakfast was also higher (89 vs. 79, *p* < 0.05) (Figure 1), with more Italian women consuming breakfast than Dominican women (Supplementary Table S2). In contrast, a lower percentage of participants from Italy consumed snacks between meals compared to the Dominican population (47 vs. 70, *p* < 0.005) (Figure 1) in both sexes (Supplementary Table S2). The majority of participants had lunch and dinner, with no significant differences between the two populations (Figure 1), while we observed that a significantly higher percentage of Italian than Dominican women had lunch and dinner (Supplementary Table S2).

### 3.3. Breakfast Habits in the Italian and Dominic Republic Populations

Since breakfast is the most important meal of the day [26], it is important to deeply investigate the breakfast habits of the populations. The breakfast location influences the quality of the meal, since home-cooked foods have usually a lower caloric content and are characterized by healthier properties compared to meals consumed outside of the home [27]. Interestingly, we found that the majority of participants from both populations consumed breakfast at home, with a higher percentage of Dominicans consuming breakfast at the bar/restaurant and the office compared to Italians (*p* < 0.01) (Table 2). The breakfast settings resulted in being significantly different between Italian and Dominican women when categorizing the population by sex (Supplementary Table S3).

Another important parameter that influences the healthy status is the meal timing, because the metabolism is influenced by the circadian rhythm [28]. We observed a statistically significant difference in the time of breakfast between the Italian and the Dominican population (*p* < 0.0002), which is maintained when categorizing the populations by sex (Table 3; Supplementary Table S4). Particularly, the majority of our sample population had breakfast between 7:30 and 9:00 a.m., whereas a lower percentage of Italians had breakfast after 9:00 a.m., regardless of sex (Table 3; Supplementary Table S4). 

**Figure 2:**
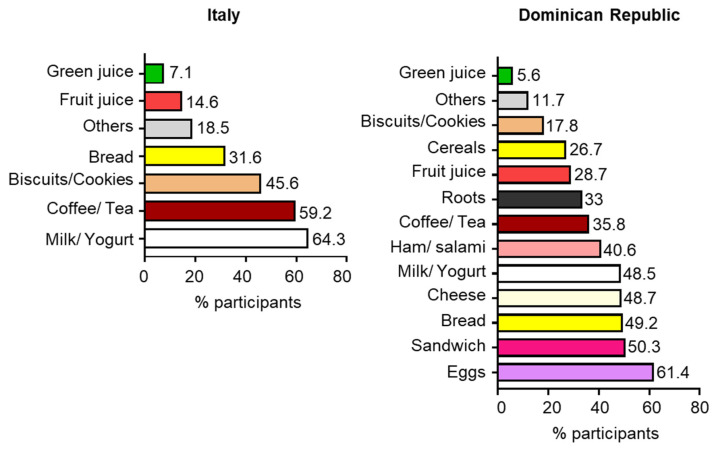
Distribution of the participants from Italy and the Dominican Republic based on food and beverage consumption for breakfast.

Cultural and environmental factors influence the choices of foods that are usually consumed for breakfast. We observed that most of the Italians had breakfast with milk/yogurt (64.3%), coffee/tea (59.2%) and biscuits/cookies (45.6%). A lower percentage of Italians consumed bread (31.6), fruit (14.6%) or green juices (7.1%) (Figure 2). Regarding the Dominican Republic population, we found that the majority of subjects had breakfast with eggs (61.4%), sandwiches (50.3%), bread (49.2%), cheese (48.7%), milk/yogurt (48.5%) and ham or salami (40.6%), whereas a smaller percentage of participants consumed coffee/tea (35.8%), roots (33%) fruit juices (28.7%), cereals (26.7%), biscuits/cookies (17.8%) and green juices (5.6%) (Figure 2).

### 3.4. Food Choices Among Italians and Dominicans for Lunch or Dinner

**Figure 3:**
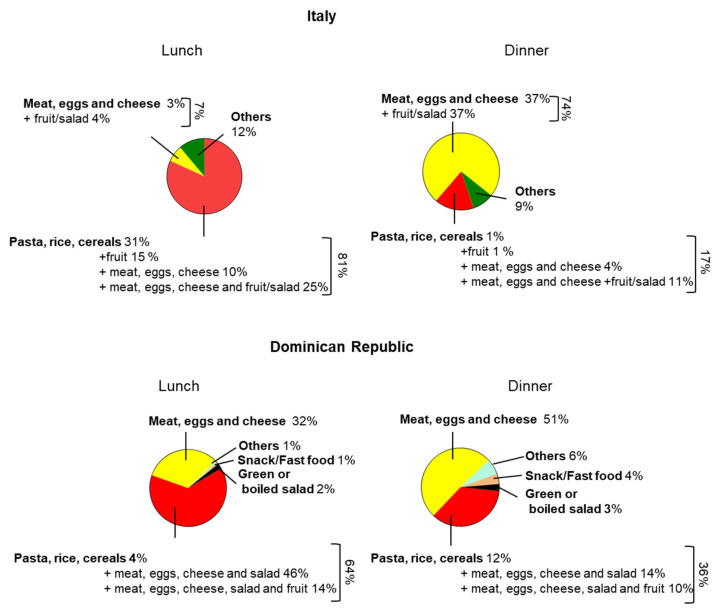
Food choices among Italians and Dominicans for lunch and dinner.

To evaluate the food choices of our participants at the main meals, we considered the consumption of different options for lunch and dinner, as shown in Figure 3. We found that 81% of Italians have a meal consisting of a first course (pasta, rice and cereals) alone (31%) or with fruits (15%), proteins (10%) or proteins with fruit (25%) for lunch, whereas 64% of Dominicans have a meal composed of first course alone (4%), with proteins and salad (46%) or with proteins and fruits (14%). In contrast, 17% of Italians consume pasta, rice and cereals alone (1%) or with fruits (1%), proteins (4%) or with proteins with fruit (11%) for dinner, whereas 36% of participants from the Dominican Republic have a meal composed of a first course alone (12%), with protein and salad (14%) or with proteins and fruits (10%) (Figure 3).

### 3.5. Percentage of Fruit and Vegetable Consumers Among Italians and Dominicans 

A significantly higher percentage of Dominicans consumed fruit juice for breakfast (28.7 vs. 14.6, *p* = 0.02), while more Italians ate fruits or vegetables in the main meals (lunch: 53.9 vs. 12.7, *p* < 0.00001; dinner: 53.2 vs. 9.4, *p* < 0.00001) (Table 4). The percentage of participants consuming FVs categorized by meals and sex can be seen in Supplementary Table S5.

Recently, it has been reported that the FV intake can be estimated through the evaluation of the skin carotenoid content by the Veggie Meter^®^ [29,30]. However, it is worthwhile to note that the skin carotenoid score is affected by several lifestyle habits, including smoking [31]. Consistent with the data presented in the literature [32], the carotenoid score was significantly higher in non-smoker participants in both populations (Italy: 347.47 ± 93.2 vs. 326.45 ± 88.9, *p* = 0.02; Dominican Republic: 286.2 ± 91.5 vs. 245.8 ± 66, *p* = *0.01*). This carries clinical significance, as previous studies have found that carotenoids might be harmful in smokers due to changes in the cell oxidative status [33]. Furthermore, our previous study revealed that the skin carotenoid content was higher in Italians when compared to Dominicans [19]. Accordingly, the carotenoid score of smokers and non-smokers was significantly higher in the Italian population (smokers: 326.45 ± 88.9 vs. 245.8 ± 66, *p* < 0.0001; non-smokers: 347.47 ± 93.2 vs. 286.2 ± 91.5, *p* < 0.0001) (Table 5) in both sexes (Supplementary Table S6).

### 3.6. Association Between Carotenoid Score and Different Variables

The results of the multiple linear regression analysis are shown in Table 6. The carotenoid score resulted in being positively associated with sex (*p* = 0.009), age (*p* = 0.001), vegetable consumption (*p* = 0.041) and the perception of a healthy diet (*p* = 0.001) in the Dominican population, while the carotenoid score was associated with sex (*p* < 0.0001) and both vegetable (*p* = 0.0008) and fruit (*p* < 0.0001) consumption in the Italian population. 

## 4. Discussion

This study was the first to compare the general characteristics and eating habits focusing on FV intake and its association with the skin carotenoid content between two population samples from both a Mediterranean country and a non-Mediterranean country, represented by Southern Italy and the Dominican Republic, respectively. 

Differences between the Italian and Caribbean populations in general are mostly deductible. Among the anthropometric and general characteristics of our population sample, the BMI values were significantly higher in the Dominican population. These findings are consistent with the high age-standardized prevalence of overweight among Dominican adults of both sexes as estimated by the World Health Organization (WHO) in 2022 [34]. In contrast, the percentage of smokers was lower in the Dominican Republic than in Italy, whose data worsened compared to those previously reported in the population of the same geographical area [24]. Notably, more than 80% of the Italian participants declared consuming iodized salt compared to approximatively 70% of Dominicans, confirming the success of a persistent campaign of prophylaxis carried out in the Southern Italian region in the last decades, which resulted in an increased salt iodine intake along with the reduced goiter prevalence in the population [35,36,37] and the achievement of iodine sufficiency [35,36,38]. Furthermore, while most participants from both samples perceived that they have a healthy diet in terms of the principles guiding current nutrition recommendations and general lifestyle behaviors, several differences between the Italian and the Dominican Republic populations were identified, specifically in the frequency of meals, the time settings and the food groups reported to be ingested daily. In particular, the percentage of Italian participants who reported eating five meals and having breakfast was higher than the Dominicans, who reported snacking between meals more frequently. 

Several studies suggest an inverse relationship between breakfast consumption and a higher risk of overweight and abdominal obesity [39,40,41]. Aside from the habit of eating breakfast, its quality has implications for daily energy, body weight changes and metabolic and chronic disease risk through a variety of mechanisms.

In our study, we observed that most of the participants of both populations had breakfast at home and only a small percentage consumed it at a bar or the office. The habit of eating breakfast at home is beneficial, since meals eaten outside of the home tend to have larger portions, have a higher caloric content and are usually unhealthier food choices when compared to home-cooked meals [27].

Regarding the time of breakfast, we found that it is influenced by the nationality. Indeed, although most of both population samples started eating before 9:00, a higher percentage of Dominicans had breakfast later in the morning. However, the food choices remained unchanged in the three different time ranges analyzed, with Dominicans having breakfasts which consisted of eggs, sandwiches, bread, cheese, milk/yogurt, ham or salami, coffee/tea, roots and fruit juice, while the Italians preferred milk/yogurt, coffee/tea, biscuits/cookies and bread. Notably, breakfast is commonly considered as the most important meal of the day, including preferential choices from three basic foods groups, dairy products, cereals and fruits, to provide the energy to start the day [42]. 

Since eating habits associated with individuals’ lifestyle behaviors are shaped by society and the cultural contexts of their lives, an ideal diet, such as the MD, should be adapted to populations around the world. In this context, comparing the dietary habits of non-Mediterranean and Mediterranean countries is worthwhile to develop guidelines for a healthy lifestyle based on the MD recommendation. To the best of our knowledge, there are only a few studies evaluating the dietary habits of Italian and Latino populations. In particular, Ojeda-Granados et al. performed a cross-sectional study investigating the dietary choices of Italian women from Southern Italy and Western Mexico [43]. They observed that both populations follow a healthy diet based on the MD or the traditional Mexican diet. However, the Mexican women lacked in the consumption of several traditional Mexican diet foods. In another study, the dietary behaviors of adults from Cuba were compared to Italian ones, demonstrating that Cuban adults have less restrained eating than Italians [44]. Specifically, by applying the concept of the “Planeterranean” diet globally, it is recommended to build country-specific pyramids based on locally available foods, which have the same healthy nutritional properties as the MD [45]. The MD is primarily a plant-based dietary pattern that includes the daily consumption of several portions of FVs as a peculiar characteristic of all dietary indices evaluating the level of adherence to the MD [46].

We have largely investigated the adherence to the MD in the Mediterranean region of Southern Italy, and we found a moderate adherence to the MD in both adolescent [47,48] and adult population samples [24]. In contrast, adults in the Dominican Republic had a lower MD adherence score compared to the Italian population, even after age-adjusting data and categorizing by sex [19]. Specifically, data from the inhabitants of Southern Italy and the Dominican Republic regarding the FV intake showed that most of each population’s percentage distribution relative to the cut-off points was outside of the recommendations according to the MEDAS [19].

According to the WHO guidelines, the recommended consumption of FVs is at least 400 g/day [49]. The adequate consumption of FVs, rich in vitamins (e.g., folate, pro-vitamin A and vitamin C), minerals (e.g., calcium, magnesium and potassium), phytochemicals (e.g., carotenoids, flavonoids and phenolics) and fibers, reduces the risk of cardiovascular diseases, diabetes mellitus and stomach and colorectal cancers. Data from the WHO indicate that approximately 16 million (1.0%) disability-adjusted life years and 1.7 million (2.8%) deaths worldwide are attributable to low FV consumption [49]. 

Results from a systematic review, which aimed to evaluate the differences in dietary intakes of ethnic groups worldwide, reported that Black/African American groups are the poorest consumers of FVs [50]. Specifically, in the United States, Black groups reported consuming significantly fewer vegetables compared to White and Hispanic groups, while Hispanic and Latino groups had higher fruit intakes than White groups [50]. There is compelling evidence that the key factors that influence the purchase and consumption of these foods are the high cost of FVs with respect to other foods and/or limited access to them, as well as the widespread availability of unhealthy choices, such as fast foods with a high energy content, which are associated with chronic disease risk and death [51,52]. 

Despite the benefits of consuming FVs, we found that most participants of both populations did not prefer this choice for breakfast, as previously discussed. However, approximately half of the Italian population consumed FVs in the main meals, whereas the Dominicans declared this choice mainly at lunch. Confirming this different eating habit, we have previously reported that the average of the skin carotenoid values, measured by the Veggie Meter^®^ spectroscopy device, was greater in the Italian population than the Dominican population [19]. Here, we evidenced that, by classifying both populations on smoking status, higher carotenoid scores were detected in the Italian non-smoker population. These findings are in line with the data described in a recent narrative review, in which the smoking status emerged as a factor inversely related to skin carotenoid levels [32]. Moreover, there are several other factors that may affect skin carotenoid levels which must be identified and considered to optimize their levels along with the FV intake among diverse populations. Our results showed, in a multiple regression analysis, a direct correlation between carotenoids and age, sex, vegetable consumption and the perception of having a healthy diet in the Dominican Republic population. Notably, skin carotenoid levels were also correlated with age and sex, but also with FV intake, in the Italian sample.

Regarding age and sex, conflicting data exist with respect to the ability of these two biological factors to influence skin carotenoid levels in adults [53,54,55], suggesting that further research may be required to better characterize their potential influence on carotenoid levels. Furthermore, it is worthwhile to note that some factors, such as racial/ethnic backgrounds, genetics, recent sun exposure along with the type of spectroscopy device used for performing measurements, may impact the associations between the abovementioned factors and skin carotenoids.

Even though the present study provides valuable information, it has some limitations. The data were collected in a few study sites and may not be representative of the general population of both countries. Additionally, even though skin carotenoid levels are indicative of the serum carotenoid concentration, they might differ due to several factors, including their metabolism and tissue distribution.

## 5. Conclusions

To the best of our knowledge, this is the first study assessing the dietary habits and comparing the skin carotenoid levels between two adult populations from a Mediterranean country and a non-Mediterranean country, represented by Italy and the Dominican Republic, respectively. As expected, we found different eating habits and food choices in the two populations, indicating that the cultural and social environment influences the dietary pattern. Interestingly, the healthier eating model observed in the Italian population compared to the Dominican Republic population suggested that, even if the adherence to the MD pattern is decreasing in the Mediterranean area, there is still a greater awareness in Italy. Considering the importance of FV intake in preventing several chronic diseases, our findings confirm the need to promote nutrition-based policies, programs and interventions to encourage FV intake worldwide. The information provided in our study can help in the planning of nutritional public health strategies and can aid in the adaptation of the eating habits, especially for the Dominican population, towards a better dietary model like the MD pattern.

## Figures and Tables

**Table 1:** Anthropometric and general characteristics of our study population.

Characteristics	Italy	Dominican Republic	*p*-Value
Subjects (n)	601	394	
BMI (kg/m^2^), mean ± SD	22.7 ± 3.8	25.1 ± 4.7	0.0001
Underweight (%)	6.15	3.8	
Normal weight (%)	64.45	49	ns
Overweight (%)	23.42	32.9	
Grade 1 obesity (%)	4.82	10.2	
Grade 2 obesity (%)	1.50	4.1	
Smokers (%)	24.8	8.1	0.001
Frequency of biochemistry analysis > 12 months (%)	75.7	49.7	0.0001
Periodic control of arterial pressure (%)	33.6	24.4	ns
Iodine salt intake (%)	83.2	69.3	0.02
Having a healthy diet (%)	62.7	51.5	ns

BMI: body mass index; ns: not significant.

**Table 2:** Breakfast settings.

	Italy	Dominican Republic	*p*-Value
Home (%)	95.5	82.6	0.01
Bar/Restaurant (%)	2.6	6.5	
Office (%)	1.9	10.9	

**Table 3:** Time of breakfast.

	Italy	Dominican Republic	*p*-Value
6.00–7.30 a.m. (%)	34.9	27.1	0.0002
7.30–9.00 a.m. (%)	59.3	45.4	
After 9.00 a.m. (%)	5.8	27.5	

**Table 4:** Percentage of participants consuming fruits and vegetables categorized in eating events among Italians and Dominicans.

Meal	Italy	Dominican Republic	*p*-Value
Breakfast (%)	14.6	28.7	0.02
Snacking between two meals (%)	30.9	32.7	ns
Lunch (%)	53.9	12.7	<0.00001
Dinner (%)	53.2	9.4	<0.00001

ns: not significant.

**Table 5:** Carotenoid scores in the Italian and Dominican Republic populations categorized by the smoking status.

	Italy	Dominican Republic	
	Carotenoid Score (Mean ± SD)		*p*-Value
Smokers	326.45 ± 88.9	245.8 ± 66	<0.0001
Non-smokers	347.47 ± 93.2	286.2 ± 91.5	<0.0001
*p*-Value	0.02	0.01	

**Table 6:** Multiple regression analysis among carotenoid scores and different variables in the Italian and Dominican Republic populations.

	Italy		Dominican Republic	
Variable	β (95% CI)	*p*-Value	β (95% CI)	*p*-Value
Sex	54.97 (40.48, 69.46)	<0.0001	0.078 (0.02, 0.14)	0.009
Age	0.15 (0.46, 0.75)	0.63	0.008 (0.005, 0.010)	0.001
BMI	−1.17 (−3.19, 0.77)	0.24	−0.01 (−0.01, 0.001)	0.08
Vegetable consumption	25.42 (10.61, 40.23)	0.0008	0.12 (0.005, 0.24)	0.041
Fruit consumption	38.61 (20.14, 57.09)	<0.0001	0.04 (−0.09, 0.17)	0.56
Healthy diet	−13.28 (−28.31, 1.743)	0.08	0.12 (0.07, 0.18)	0.001
Adj.R2	0.13		0.14	

Adj: adjusted; BMI: body mass index; CI: confidence interval.

## Data Availability

The original contributions presented in the study are included in the article/Supplementary Materials, further inquiries can be directed to the corresponding author.

## Supplementary Materials

The following supporting information can be downloaded at: https://www.mdpi.com/article/10.3390/foods13203323/s1, Table S1: Anthropometric and general characteristics of our study populations categorized by sex; Table S2: Distribution of the participants categorized by sex from Italy and the Dominican Republic based on their eating occasions; Table S3: Breakfast settings in the population sample categorized by sex; Table S4: Time of breakfast in the population sample categorized by sex; Table S5: Percentage of participants consuming fruits and vegetables categorized in eating events among Italians and Dominicans divided by sex; Table S6: Carotenoid score in the Italian and Dominican Republic populations categorized by the smoking status and sex.
