# Supplementary material for: Comparing the Dietary Habits and the Food Choices Between Italian and Dominican Adult Populations: Focus on Fruit and Vegetable Intakes and Their Association with Skin Carotenoid Levels
Source: Foods. 2024 Oct 19;13(20):3323. doi: 10.3390/foods13203323 (PMC11508049; doi:10.3390/foods13203323)
Supplement: Supplementary file 1 [file foods-13-03323-s001.zip › foods-3206672-supplementary.pdf]

Supplementary Table S1. Anthropometric and general characteristics of our study population categorized by sex.

Characteristics	Italy	Dominican Republic	*p*-Value
Subjects (n)	W: 331<br>M: 270	W: 213<br>M: 181	
BMI (kg/m^2) (Mean ± SD)	W: 21.8±3.35<br>M: 23.9±3.9	W: 25.2±5.2<br>M: 24.9±4.1	W: 0.0001<br>M: 0.009
Underweight (%)	W: 12.7<br>M: 4.4	W: 4.2<br>M: 3.3	W: 0.00002<br>M: ns
Normal weight (%)	W: 73.1<br>M: 63.3	W: 49.3<br>M: 48.6	
Overweight (%)	W: 11.2<br>M: 24.4	W: 31.0<br>M: 35.4	
Grade 1 obesity (%)	W: 2.4<br>M: 5.9	W: 8.5<br>M: 12.2	
Grade 2 obesity (%)	W: 0.6<br>M: 1.8	W: 7.0<br>M: 0.6	
Smokers (%)	W: 25.4<br>M: 34.1	W: 5.6<br>M: 11.1	W: 0.0002<br>M: 0.0001
Frequency of biochemistry analysis >12 months (%)	W:78.8<br>M: 73.7	W 41.3<br>M: 59.7	W: 0.0001<br>M: 0.03
Periodic control of arterial pressure (%)	W: 35.0<br>M: 31.8	W: 30.5<br>M: 17.1	W: ns<br>M: 0.01
Iodine Salt Intake (%)	W: 84<br>M: 82.2	W: 76.1<br>M: 61.3	W: ns<br>M: 0.001
Make a healthy diet (%)	W: 62.8<br>M: 62.6	W: 50.2<br>M: 53.0	W: ns<br>M: ns

BMI: Body Mass Index, W: woman, M: man

Supplementary Table S2. Distribution of the participants categorized by sex from Italy and Dominican Republic based on their eating occasions.

	Italy	Dominican Republic	*p*-Value
5 Meals/day (%)	W: 50.8<br>M: 34.1	W: 23<br>M: 28.2	W: 0.00004<br>M: ns
Eat breakfast (%)	W: 91.5<br>M: 86	W: 79.3<br>M: 77.3	W: 0.02<br>M: ns
Habits of snacking between meals (%)	W: 54.5<br>M:38.9	W: 69<br>M:71.8	W: 0 .001<br>M: < 0.00001
Lunch (%)	W: 99.9<br>M:98.9	W: 93.4<br>M:95	W: 0.03<br>M: ns
Dinner (%)	W: 99.4<br>M: 98.5	W: 92.5<br>M:98.3	W: 0.03<br>M: ns

W: woman, M: man

Supplementary Table S3. Breakfast settings in the population sample categorized by sex.

	Italy	Dominican Republic	*p*-Value
Home (%)	W: 95.2<br>M: 91.5	W: 82.2<br>M: 83	W: 0.008<br>M: ns
Bar/Restaurant (%)	W: 3.3<br>M: 5.5	W: 4.7<br>M: 8.5	
Office (%)	W: 1.5<br>M: 3	W: 13.0<br>M: 8.5	

W: woman, M: man

Supplementary Table S4. Time of breakfast in the population sample categorized by sex.

	Italy	Dominican Republic	*p*-Value
6.00-7.30 am (%)	W: 37<br>M: 32.3	W: 24.2<br>M: 30.5	W: 0.00008<br>M: 0.0003
7.30-9.00 am (%)	W: 58.1<br>M: 60.8	W:48.5<br>M:41.6	
After 9.00 am (%)	W: 4.9<br>M: 6.9	W: 27.2<br>M: 27.7	

W: woman, M: man

Supplementary Table S5. Percentage of participants consuming fruits and vegetables categorized in eating events among Italians and Dominicans divided by sex.

Meal	Italy	Dominican Republic	*p*-Value
Breakfast (%)	W: 11.5	W: 26.3	0.006
	M: 7.3	M: 31.5	0.00001
Snacking between two meals (%)	W: 41.7	W: 37.6	ns
	M:29.5	M: 27.1	ns
Lunch (%)	W: 46.4	W: 11.7	< 0.00001
	M: 61.8	M: 13.8	< 0.00001
Dinner (%)	W: 48	W: 7.9	< 0.00001
	M: 59.5	M: 11.1	< 0.00001

W: woman, M: man

Supplementary Table S6. Carotenoid score in the Italian and Dominican Republic population categorized by the smoking status and sex.

	Italy	Dominican Republic	
	Carotenoid score (Mean ± SD)		*p*-Value
Smokers	W: 342.36±92.47<br>M: 343.45±93.3	W: 226.4±36.2<br>M: 257.5±77.3	W: < 0.0001<br>M: 0.0002
No-smokers	W: 341.96±92.38<br>M: 342.17±92.37	W: 280.3±93.3<br>M: 293.5±88.8	W: < 0.0001<br>M: < 0.0001

W: woman, M: man
